# Biomimetic Random Pulse Computation or Why Do Humans Play Basketball Better than Robots?

**DOI:** 10.3390/biomimetics8080594

**Published:** 2023-12-07

**Authors:** Mario Stipčević

**Affiliations:** Photonics and Quantum Optics Unit, Center of Excellence for Advanced Materials and Sensing Devices, Ruđer Bošković Institute, Bijenička Cesta 54, 10000 Zagreb, Croatia; mario.stipcevic@irb.hr

**Keywords:** biomimetic computing, random pulse computing, stochastic computing, biomimetic robot, neuronal computing, biologically inspired computing, prosthetics

## Abstract

In this work, we compare the basketball scoring performance of two imaginary (simulated) mechanical robots in conditions of erroneous information-processing circuits: Machine, whose moves are controlled by a conventional digital computer and Man, controlled by a random pulse computer composed of biologically-inspired circuits which execute basic arithmetic operations. This is the first comparative study of robustness of the digital and the random pulse computing paradigms, with respect to the error rate of the information-processing circuits ($p_{err}$), for a mechanical robot. In spite of the fact that Man’s computer consists of only about 100 logic gates while Machine’s requires about 3500 gates, Man achieves a significantly higher scoring probability for $p_{err}$ in the range from 0.01% all the way to 10%, while at lower $p_{err}$, both converge to the perfect score. Furthermore, Man’s hits make up a smooth Gaussian distribution with a vanishing probability of making large misses even at the highest $p_{err}$, while Machine is prone to spectacular misses already at $p_{err}$ as low as 1 part-per-million. These findings indicate that the biologically inspired computation requires less hardware for the same task, and ensures higher robustness and better behaving operation than digital computation, which are characteristics of importance for the survivability of living beings.

## 1. Introduction

Humans are probably the only living beings capable of logical thinking, that is inventing, memorizing, recalling, communicating, learning and performing complex algorithms based on yes/no decisions. As such, algorithmic thinking is formalized in the so-called Turing computational paradigm, which can be conveniently described by Boolean algebra. A recipe for making an apple pie is one example of an algorithm. Others include: growing wheat, making bricks and houses, driving a car, etc. Some are purely mental, oriented to information processing, such as the algorithm for multiplication of two long decimal numbers or finding the largest common divisor of two integers, and so on.

Throughout its history, mankind strived to make machines that could perform algorithms quicker, with less effort and with fewer errors than humans do. Regarding the latter, various technologies evolved with time and today we have machines that almost think. The first machines that improved on human mental capacity were computing machines made of mechanical parts, such as the Stepped Reckoner of Leibniz, which he successfully assembled in 1672. It was a mechanical calculator that could add, subtract, multiply and divide. Further advances in computing machines were made using technologies of electro-mechanical switches (relays) [1] and electron tubes [2], both having drawbacks of a high power consumption and large physical dimensions and weight, as well as the problem of fast wear of the switching components, which led to short maintenance periods. The first transistor-based computers that appeared in the mid 1950s offered far less power consumption, a lower failure rate and greater speed. This created the idea of integrating many transistors, forming Boolean logic circuits, on a single slice of silicon crystal: the so-called chip. The rest is history. The invention of an integrated microprocessor was based upon von Neumann architecture in the early 1970s, and the steady exponential increase in information-processing speed of microprocessors (Moore’s Law) led to their widespread use in all information-processing and communication devices, such as in personal computers, smart phones, home appliances, cars, medical equipment, etc.

However, it has been noted that even powerful digital microprocessors had difficulties in performing tasks that are simple and natural to humans, such as picture and speech recognition, or learning [3]. This should not come as a surprise because the brain internally operates upon a completely different computational paradigm which encodes information in time series of short electrical impulses, rather than in the logical states. The apparent logical behavior of humans is possible because our brains contain certain regions specialized for a two-way conversion between the internal stream of impulses and the apparent logical actions. For example, a child performing the pen-and-pencil algorithm for the addition of two multi-digit numbers needs to convert a multitude of optical information into an abstract idea of a digit, and then to recall the rule for adding digits—do the math—and finally output the correct digit by sending a myriad of pulses to the muscles of a hand that would write it out. On the other hand, if a digital computer needed to process a huge input of visual data streaming in from a camera in order to recognize that a suitably shaped wooden piece, or a banana, waved in the hand of another child swirling around at a full speed, in fact represents a toy gun, it would be in a deep trouble. But, for a child, it is a child’s game. One could hypothesize that each of these computing paradigms (Turing and impulse) have their own strong advantages over the other [4].

To investigate that hypothesis, in this work we investigate one exemplary use of the random pulse computation (RPC) paradigm, using a simple model which is by no means meant to be a realistic model of human brain, but which is inspired by information-processing components found in living neurons. In particular, we study and compare the performance of two imaginary basketball-playing robots: a robot named “Man” that operates upon the biologically inspired RPC paradigm and a robot named “Machine” that operates upon the conventional digital computation (DC).

Previous art was focused on the investigation of immunity of the information processing against the noise present in the input data. That includes: contour edge recognition in images [4,5,6,7], image gamma correction [8], pattern/symbol recognition [9] and speaker/sound localization [10]. While in these works a superior noise immunity of the RPC paradigm has been found, in this study we are up to a completely different notion of immunity, namely the one related to the hardware damage. There are three differences to the previous art.

First, in previous studies, noise was added to the input data while the information-processing circuitry was operating flawlessly, whereas here we assume that the data are noiseless, but that the information-processing circuits are subject to a random failure. The second difference is that, in the previous art, a single instance of performing a task, such as the processing of the same image with DC and RPC, can clearly display all of the major differences between the two paradigms. However, a single instance of two robots performing the same task tells us virtually nothing: one needs to perform a large number of trials in order to deduce the statistical distribution of outcomes. And finally, in the previous art, a single statistical imperfection, for example on a small part of the picture, does not affect the overall impression and evaluation of the result, because it is an average performance that matters. The fundamental difference with the performance of a mechanical robot is that a single wrong move, for example of a warehouse picking robot, or a surgical robot, can cause irreparable damage. Therefore, in the proposed research, we are much more interested in the severity and overall probability of outliers than in the average behavior.

To the best of our knowledge, this work is the first comparative study of robustness of the system for controlling movements of a mechanical robot. The motivation for this line of research is twofold. Firstly, when choosing a robot technology for a critical mission, it is important to know which of the two computing paradigms would offer an advantage in the case of operation in a harsh environment, such as in a highly corrosive, high-temperature or strong radiation environment, which could cause either soft or permanent damage to the functionality of its information-processing circuits. This is relevant for the survivability of the robot. Secondly, this line of research could give us an insight into the following question: why did living beings, which are indeed composed of intrinsically unreliable components, evolve to use impulse instead of digital computation?

The paper is organized as follows. First, the random pulse computation paradigm is briefly explained and all computational circuits used in this study are presented. Next, the physical model of basketball shooting is developed, which results in a mathematical function describing the ballistic of the hit. The Methods section presents the implementation of required computation in both RPC and DC paradigms. What follows are a presentation and comparison of results of computer simulations of RPC and DC approaches, followed by concluding remarks.

## 2. Random Pulse Computation Paradigm

Contemporary digital computers are based upon the Turing machine theoretical model realized in the von Neumann architecture. Each number, in the digital computing paradigm (DC), is represented by a string of binary digits stored in the computer’s memory. Elementary arithmetic operations—subtraction, addition, multiplication and division—are performed by manipulation of the binary digits in a fashion similar to pen-and-pencil algorithms taught to children in elementary schools. The actual algorithms are based upon Boolean algebra logic operations and executed by so-called logic gates (OR, AND, NOT), while memory is realized by a sequential logic circuit, so-called flip-flop, which in turn is made of logic gates. These arithmetic algorithms, optimized for a fast execution, are implemented as a complex circuit containing thousands of logic gates, known as arithmetic logic units (ALUs), which are an essential part of the von Neumann architecture of a digital computer.

**Figure 1:**
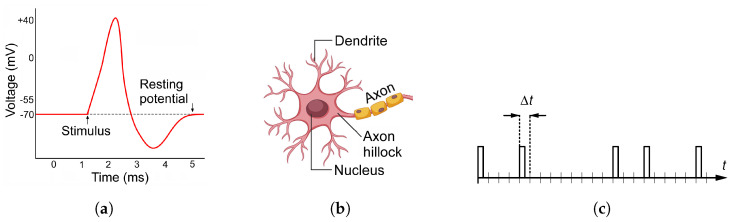
A typical neuronal pulse (**a**); schematic drawing of a neuron (**b**); and a random pulse train model used in the random pulse computing paradigm (**c**).

But, von Neumann is not only known for his work on digital computers: he was also the first to propose biologically inspired impulse-counting computation in 1956 [11,12] followed by the works of others [13,14,15]. Namely, in the human brain, information between neurons is represented and exchanged via short electrical pulses, such as the one schematically shown in Figure 1a. A typical neuron, as illustrated in Figure 1, has one output and many (up to thousands) inputs. Neurons are mutually interconnected via dendrites (their inputs) and axons (their outputs) into a complex neural network. It has been established that a typical neuron features dendrites which upon receipt of a pulse release either a small amount of positive charge (excitatory pulses) or negative charge (inhibitory pulses) into the neuron cell. All of the charge received by a neuron cell is summed up in the so-called axon hillock, thus establishing its electric potential. When the potential reaches a certain threshold, the axon hillock releases a nerve pulse into the axon (output of the neuron) and resets its potential back to the resting level of about −70 mV. The pulse is conveyed to the input(s) of other connected neuron(s). This operation of the axon hillock is logically equivalent to the operation of a bidirectional (up/down) counter, a circuit well-known in digital engineering [16], for which the state is additionally compared to a predefined threshold value. Such a counter advances by one upon every pulse that arrives on its Up input, and counts down by one upon each pulse arriving at its Down input. Such a circuit is indeed used in RPC.

The neuronal pulse cycle lasts about 4–5 ms; therefore, the maximum pulse rate is limited to about 200–250 cps. Neurons communicate to each other via series of pulses which appear to be distributed randomly over time [17]. Such a phenomenon is called a random pulse train (RPT). In the random pulse computation, we model an RPT by a sequence of electric pulses that can appear in an uninterrupted series of time intervals of duration Δt, as shown in Figure 1c. Each individual pulse is generated as a Binomial event with probability p∈[0,1]. Such an RPT represents the real number *p*. For example, if, for each time interval, a fair coin is tossed and pulse generated if the coin shows heads, then such an RPT represents the number 1/2. If an RPT is generated by tossing a heavily unfair coin which yields heads with probability of, say, 1/3, then it will represent number 1/3, etc. Usually, pulses are assumed to be the digital logic pulses, because they can be easily generated, manipulated and analyzed by Boolean logic circuits conveniently available in reconfigurable chips such as Field Programmable Gate Array (FPGA), but other technologies have also been investigated [18].

**Figure 2:**
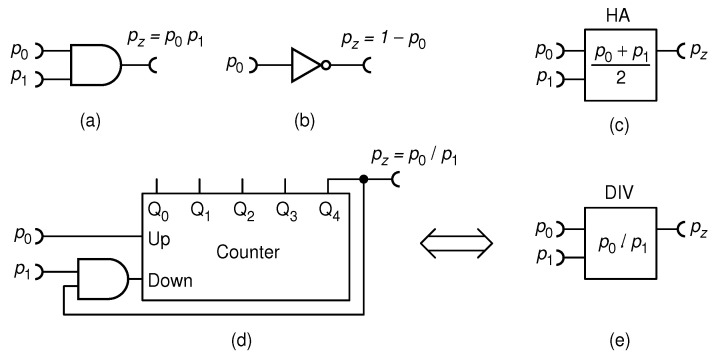
Elementary operations circuits of a RPC: multiplier (**a**); inverter (**b**); half-adder (symbol) (**c**); divider (**d**); and the divider symbol (**e**).

As opposed to the digital computer which relies on ALU, a complex circuit consisting of thousands of logic gates, the elementary arithmetic operations in RPC can be performed with much less hardware. The RPC circuits used in this study are shown in Figure 2.

For example, given two statistically independent RPTs with pulse probabilities $p_{0}$ and $p_{1}$, the probability to have simultaneous impulses form both is simply a product of the two pulse probabilities, $p_{0}p_{1}$, as guaranteed by the law of probability. Incidentally, the Boolean operation X AND Y yields a pulse if and only if both A and B carry a pulse at the same time. Thus, a single AND gate can multiply two numbers, as shown in Figure 2a. It can be shown that the NOT gate calculates the function $f\left(p_{0}\right)=1-p_{0}$, as shown in Figure 2b. Plain addition of numbers cannot be performed, since the sum of two probabilities, say $p_{0}$ and $p_{1}$, spans between 0 and 2, which cannot be represented by a probability. The solution is found in the half-adder circuit (HA), shown schematically in Figure 2c, which calculates $(p_{0}+p_{1})/2$. The division can be effectively made by the use of the up/down counter, mentioned before, as shown in Figure 2d. Interestingly, equivalents of Boolean operations needed for realization of this and other RPC circuits are found in the synapses of biological neurons [19]. This particular divider circuit, proposed in our previous research [20], is quite precise, fast and well-suited for the purpose of this study. Henceforth, it will be represented by a symbol shown in Figure 2e. A detailed construction of the half-adder and a review of other known RPC circuits can be found elsewhere [4,20].

## 3. Physical Model

In order to compare the performance of the two computing paradigms, we model two robots: Man, which makes use of the random pulse computer, and Machine, which makes use of the standard digital processing used in virtually all contemporary robots.

**Figure 3:**
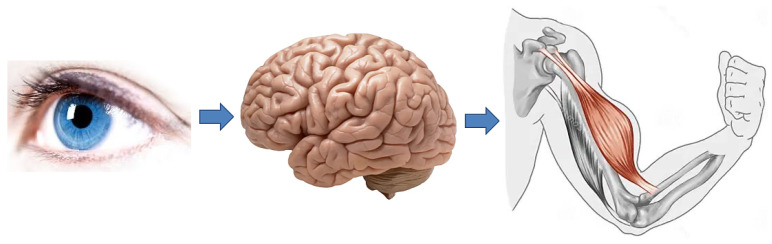
General architecture of a basketball playing robot. Visual information is used to dynamically distill distance *D* to the hoop at game time. Through training, the CPU (robotic brain) should have acquired the other necessary ballistic quantities beforehand, such as: the ball launching length *s*, height of the hoop with respect to the player Δh, and launching angle ϑ with respect to the horizontal plane. This allows the CPU to compute force $f_{H}$ by which the hand has to push the ball in order to score a point.

The two basketball-playing robots, Man and Machine, could be fairly complex: they should have some kind of vision and possibly other sensors that would pour large amounts of information into their central processing unit (CPU), or robotic brain. The output of the CPU should empower various actuators that would create required movements, notably those responsible for shooting the ball into the hoop. This general architecture of a basketball-playing robot is schematically shown in Figure 3.

**Figure 4:**
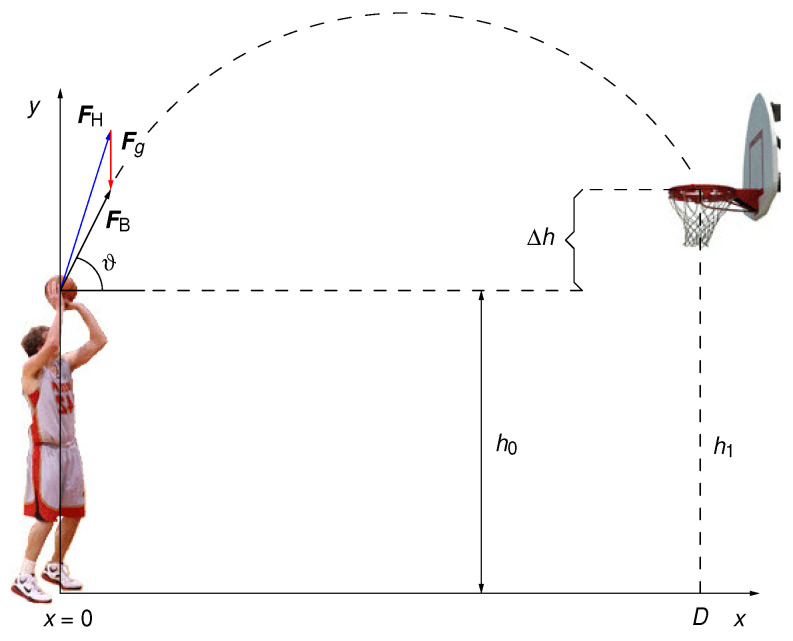
Forces, distances and the reference frame used in the modeling of a basketball player. There are two forces that act upon the ball: force exerted by hand ($\vec{F_{H}}$) and gravitational force ($\vec{F_{g}}$). The resulting net force acting upon the ball ($\vec{F_{B}}$) makes an angle (ϑ) with respect to the horizontal axis, which we call the “launch angle”.

Modeling of the robots in this study will be limited to the ballistic part of the information processing, illustrated in Figure 4.

The total force $\vec{F_{B}}$ acting on the ball of mass *m* is the vector sum of the force exerted by the player’s hand ($\vec{F_{H}}$) and the gravitational force $F_{g}$:

(1) $${\vec{F}}_{B}={\vec{F}}_{H}+{\vec{F}}_{g}$$

where:

(2) $${\vec{F}}_{B}=F_{B}\cos\left(\vartheta \right)\hat{x}+F_{B}\sin\left(\vartheta \right)\hat{y}$$

(3) $${\vec{F}}_{g}=-mg\hat{y}.$$

Combining Equations (1)–(3), one obtains:

(4) $$F_{B}=mg\left(-sin\left(\vartheta \right)+\sqrt{{\frac{F_{H}}{mg}}^{2}-cos^{2}\left(\vartheta \right)}\right)$$

Solving Equation (4) for $F_{H}$ yields:

(5) $$F_{H}=F_{B}\sqrt{1+2\frac{mg}{F_{B}}sin\left(\vartheta \right)+{\left(\frac{mg}{F_{B}}\right)}^{2}}.$$

Since, in a realistic case, the hand force $F_{H}$ is about an order of magnitude greater than the ball weight (mg), the following expression holds to an excellent approximation:

(6) $$F_{H}=F_{B}+mg\sin\left(\vartheta \right).$$

Initially, the ball is at rest. It is then accelerated by the hand on a path of length *s*, tilted at an angle ϑ with respect to the horizontal axis, with a constant force $F_{B}$, reaching velocity $v_{0}$ at release. The energy conservation yields:

(7) $$sF_{B}=\frac{mv_{0}^{2}}{2}.$$

The vertical component of the ball travel is described by:

(8) $$h_{1}-h_{0}=\Delta h=v_{0}\sin\left(\vartheta \right)t-\frac{g}{2}t^{2}$$

while the horizontal travel is given by:

(9) $$D=v_{0}\cos\left(\vartheta \right)t\Rightarrow t=\frac{D}{v_{0}\cos\left(\vartheta \right)}$$

where Δh is the vertical difference between the height of the hoop and the starting point of the ball, ϑ is the launch angle measured from the horizontal plane, *D* is the horizontal distance between the player and the center of the hoop and $v_{0}$ is the launch velocity of the ball. Inserting Equation (9) into Equation (8) yields:

(10) $$\Delta h=D\tan\left(\vartheta \right)-\frac{g}{2}\frac{D^{2}}{\cos^{2}\left(\vartheta \right)}\frac{1}{v_{0}^{2}}$$

and inserting Equation (7) into Equation (10) yields:

(11) $$\Delta h=D\tan\left(\vartheta \right)-\frac{g}{2}\frac{D^{2}}{\cos^{2}\left(\vartheta \right)}\frac{m}{2sF_{B}}.$$

Solving Equation (11) for $F_{B}$ gives:

(12) $$F_{B}=\frac{mg}{4s\cos(\vartheta )\sin(\vartheta )}\frac{D^{2}}{D\tan(\vartheta )-\Delta h}$$

To arrive to the required hand force, insert Equation (12) into Equation (6):

(13) $$F_{H}=\frac{mg}{4s\cos(\vartheta )\sin(\vartheta )}\frac{D^{2}}{D\tan(\vartheta )-\Delta h}+mg\sin\left(\vartheta \right).$$

Because RPC calculates only with numbers in the interval [0,1], we need to rewrite Equation (13) such that all of its terms satisfy that range

(14) $$F_{H}=mg\left(k\left(\vartheta \right)\frac{D/s}{1-\frac{\Delta h}{D}\cot\left(\vartheta \right)}+\sin\left(\vartheta \right)\right)$$

where we defined

(15) $$k\left(\vartheta \right)=\frac{1}{4\sin^{2}\left(\vartheta \right)}.$$

Finally, we arrive to:

(16) $$F_{H}=\frac{2mg}{C}\left[\frac{1}{2}\left(k\left(\vartheta \right)\frac{CD/s}{1-\frac{\Delta h}{D}\cot\left(\vartheta \right)}+C\sin\left(\vartheta \right)\right)\right]$$

where we introduced an arbitrary dimensionless scaling constant C>0. We keep the factor of 1/2 in front of the round brackets because, as mentioned before, an exact summation of two terms in RPC is only possible if divided by two [20]. All terms in the bracket are dimensionless and in the in the range of [0,1], except for D/s. The latter is greater than 1 everywhere on the basketball court, while in the 3-pointer region it reaches its maximum value of about 10. If we take C=0.1, then both summands in the bracket will be less than or equal to 1. Because the force $F_{H}$ must be positive, we conclude that the denominator in the brackets must be positive, that is:

(17) $$1-\frac{\Delta h}{D}\cot\left(\vartheta \right)>0.$$

Indeed, it is just the kinematic condition necessary for the ball to reach the plane of the hoop from above. As noted before, we take both the ball acceleration path *s* and the launching height Δh to be constants, fixed for a given player, dependent on its physical predispositions and shooting routine. Furthermore, researchers have found that the optimal ball launching angle ϑ for direct hits is about $52^{\circ }$ [21] while for bank shoots it is $54^{\circ }$ [22]. Even though we do not investigate bank shoots here, the similarity of the two angles indicates that exercising the ball launching angle of about $53^{\circ }$ is an optimal practice for both kinds of shooting. We adopt it as the unique shooting angle, and define three constants: $k_{1}=k\left(53^{\circ }\right)=0.392$, $k_{2}=\cot\left(53^{\circ }\right)=0.754$ and $k_{3}=\sin\left(53^{\circ }\right)=0.799$. Bearing in mind that the ball mass *m* and the gravitational constant *g* are constants too, this leaves the hand force a function of a single variable, namely the shooting distance *D*:

(18) $$F_{H}\left(D\right)=G\left[\frac{1}{2}\left(k_{1}\frac{CD/s}{1-\frac{\Delta h}{D}k_{2}}+Ck_{3}\right)\right]=Gp_{H}$$

where *G* is an overall multiplicative constant:

(19) $$G=\frac{2mg}{C}$$

which has the unit of force (newton), while $p_{H}$ is a dimensionless probability that needs to be calculated on-the-fly by the player, while participating in the game. The constant *G* is a motoric hardware gain factor which determines how the pulse probability $p_{H}\in \left[0,1\right]$ translates to the actual force exerted by the actuator (robotic hand). Constant *G* should be acquired by calibration, learning or other means well-known in the art of robotics [23].

## 4. Methods

The main goal of this study is to investigate how performances of the RPC and DC computing paradigms compare, when subject to the same range of hardware failure probability $p_{err}$. As a figure of merit, we use the scoring probability in a simplified basketball game in which players make a direct shot into the hoop from various distances.

To perform the study of the two computing paradigms, we simulate both robots, namely Man and Machine, on a PC computer, using a computer program that was written from scratch.

When playing a basketball game, both robots calculate the hand force $F_{H}\left(D\right)$ required to launch the ball into the basket by using Equation (18). It is assumed that each robot is supplied with a precise value of the distance *D*, which would be obtained by the visual sensor and associated data processing which we do not consider here. The motoric hardware gain *G* would likely be different for each individual robot. It is assumed to be adjusted prior to the game and that it stays constant during the game. Being just an overall multiplicative constant, it is irrelevant for this study and is not considered.

**Figure 5:**
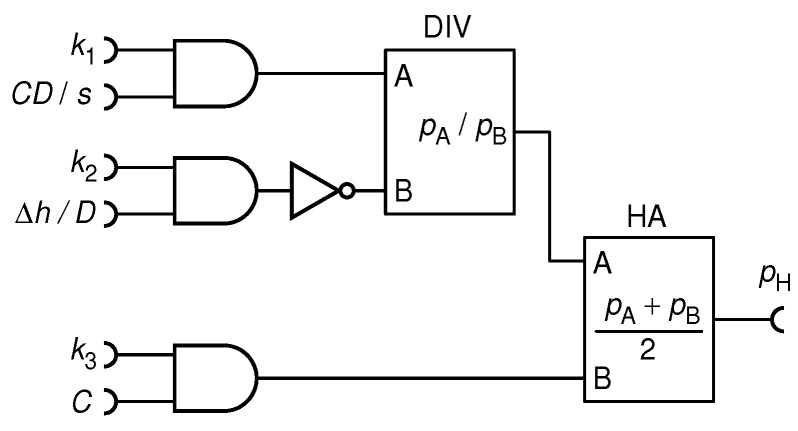
Random pulse steering circuit which implements Equation (18). It calculates hand force $F_{H}=Gp_{H}$ (up to the motoric gain *G* which is hardwired into the actuator system) which needs to be exerted on the ball, in order to hit the hoop at the distance *D*.

For its calculation of the required hand force as a function of the distance to the basket, Man uses the steering circuit shown in Figure 5. This circuit is a straightforward implementation of Equation (18) using elementary circuits listed in Figure 2 whose operation is explained in the previous section.

In our previous work, we have developed and experimentally tested successful simulation routines for the elementary RPC circuits [20], which we now use to build the kinematic processor presented here. In the simulation of this circuit, the error due to the hardware failure is implemented such that whenever a pulse appears there is a constant probability $p_{err}$, that this pulse is either deleted or that another pulse is added. It is also assumed that enough statistics from pulses are collected so that the statistical error does not dominate the calculation error due to the hardware failure, that is, the simulation calculates the probability $p_{H}$ at the output of the steering circuit. In this manner, the hardware failure can be studied and isolated from other effects.

To perform the calculation of Equation (18) for the Machine, we emulate the following arithmetic operations at the level of ALU: binary add, binary subtract, binary multiply and binary division. For the division, the Newton-Raphson method [24,25] is used. The error in the calculation of Equation (18) is implemented in the following manner. Whenever a bit is about to be manipulated, a flip of its value is implemented with a constant probability $p_{err}$. The rationale behind that is the following. Each time an individual bit is accessed in memory, there is a probability of an error, and the only possible error is a change in the stored value, namely a flip from 0 to 1 or from 1 to 0. In the modern computer hardware, such flips are extremely rare, but when they do happen they cause unpredictable behavior. In our case, it leads to a dramatic miss of the basket, as will be shown.

The simulation goes as follows. First, a distance to the hoop *D* and hardware failure probability $p_{err}$ are chosen. Using these two values, a robot calculates $F_{H}$, with which it intends to shoot the ball on the target. Now, starting from that value of $F_{H}$, the simulation program calculates the exact trajectory of the ball and finds the intersection of the trajectory of the falling ball with the plane of the hoop, which is denoted $D_{HIT}$. It can be shown that by using Equations (5) and (12), the hit distance is given by:

(20) $$D_{HIT}=\frac{v_{0}^{2}\sin\left(\vartheta \right)\cos\left(\vartheta \right)}{g}\left(1+\sqrt{1-\frac{2g\Delta h}{v_{0}^{2}\sin^{2}\left(\vartheta \right)}}\right)$$

The distance to the center of the hoop is given by:

(21) $$\Delta D=D_{HIT}-D.$$

Since the official basketball hoop diameter is $d_{H}=18$ inches and the ball diameter is $d_{B}=9.4$ inches (ball size “7”), it is taken take that a score (a successful throw) is achieved if the trajectory of the center of the falling ball intercepts the plane of the hoop within $(d_{H}-d_{B})/2=4.3$ inches (10.9 cm) from the center of the hoop. This whole process is repeated 40,000 times to gather enough statistics for a given pair $\left(D,p_{err}\right)$.

Next is a note on circuit complexity. A DC computer, for the 16 bit floating point precision, would use at least one multiplier (1461 gates), one adder/subtractor (96 gates), one divider (cca. 1700 gates) and three register memories (288 gates), totalling about 3500 gates. To evaluate Equation (20), the required computations have to be performed in a sequential order, which requires some steering logic and memory that is not counted in this estimate. On the other hand, the particular RPC circuit, in Figure 5, assuming an 8-bit counter for the divider, can be realized with only 65 logic gates plus a quantum entropy source (QES) or about 100 gates when using an 8-bit pseudo-random number generator instead of QES [20]; it requires no storage memory, and all computations are conducted simultaneously.

## 5. Results

**Figure 6:**
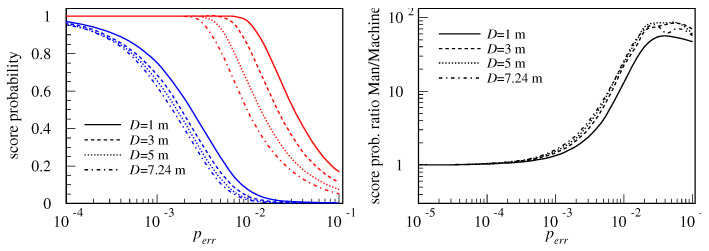
Score probability of Man (in red) and Machine (in blue) for a set of shooting distances D=1 m, 3 m, 5 m, and 7.24 m, as a function of hardware failure probability $p_{err}$ (**a**). Ratio of scoring probabilities for Man and Machine, as a function of $p_{err}$ (**b**).

First, the scoring probability is investigated for a set of shooting distances D=1 m, 3 m, 5 m and 7.24 m (the latter being the 3-pointer line). The score probabilities, as functions of the shooting distance *D* and failure probability $p_{err}$, are shown in Figure 6a. The results for Man are plotted in red and for Machine in blue. Plotted values are the result of averaging over 40,000 hits and have statistical relative errors on the order of 0.5%.

The first observation that catches the eye is that Machine always scores worse than Man. This may seem counter-intuitive, because Machine uses a deterministic computer while Man uses a probabilistic one. But, since we introduced random errors in the computation, they have both became probabilistic, and it turns out that the RPC is significantly more resilient to hardware errors than the DC. Starting from the perfect (100%) score, which they both achieve at a very low $p_{err}$, already at the error level of 0.2%, Machine’s success rate falls to 50% while Man’s is still clipped to a perfect 100% score from any shooting distance. In fact, the ratio of the scoring probability of Man to the scoring probability of Machine, shown in Figure 6b, rises as $p_{err}$ drops, reaching its maximum of about 56–84 at 4% failure probability. It is fascinating that even at 10% failure rate, Man still scores between 5.1% from the 3-pointer line and 17% from a one-meter distance, while Machine scores only from 0.09–0.36% in the same range of distances.

**Figure 7:**
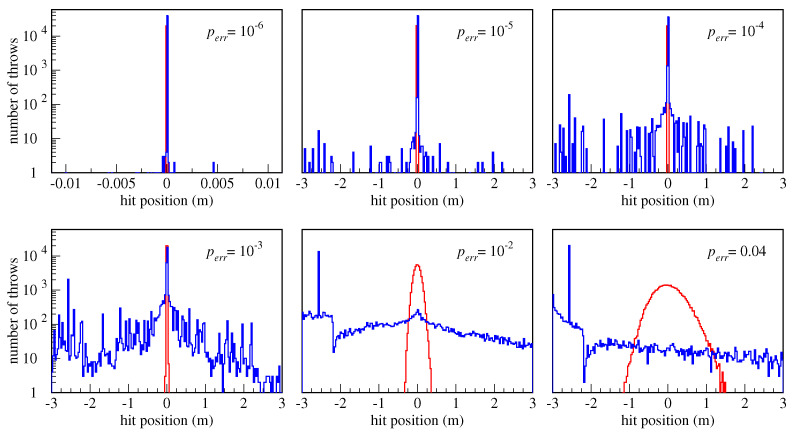
A series of plots showing hit position distributions for Man (red) and Machine (blue), at the indicated hardware error probability $p_{err}$, and shooting distance D=3 m. Each plot is generated with statistics of 40,000 shots, but not all are visible because some fall outside of the window.

A more detailed insight into the shooting patterns of Man and Machine is obtained by plotting the distributions of hit distances, shown in Figure 7. Each plot shows distributions of 40,000 hits for both robots, red for Man and blue for Machine, at the indicated $p_{err}$, and shooting distance D=3 m.

It is interesting to note that, on average, Man always scores more than or equal to what Machine scores, for any combination of *D* and $p_{err}$. However, the ratio of the distribution peaks at the center of the hoop is in favor of Machine as long as $p_{err}<0.7\%$, while for the greater $p_{err}$, Man is in advantage. This illustrates one crucial difference between the two paradigms. Given a low enough $p_{err}$, the DC is unbeatable when it comes to the precision, for example in calculating number π to a million decimal places or finding an exact solution to a numerical problem. However, when a “good enough” solution is (nearly) as good as the exact one for all practical purposes, like in this basketball game where the ball only needs to pass through the hoop, but it does not need to do it very precisely; or, in the well-known logistic “traveling salesman” problem where the lowest price itinerary is searched for. Typically, there are many solutions to this problem which are of acceptable cost. But, if any of them can be found quickly, there is no justification to spend a lot of resources searching for the best one. For such problems, the RPC approach might offer an advantage.

Another important observation is the dispersion of hits, as seen in Figure 7. At a very low hardware error probability $p_{err}=10^{-6}$, the two robots perform almost the same and both have a scoring probability near 1. Still, even at a $p_{err}$ that low, Machine features outlier hits are those which are not grouped with the main distribution peak. As $p_{err}$ grows larger, Machine shows a clear tendency to make more and more “wild” shots which miss the hoop dramatically. In the particular simulation of 40,000 hits used to generate the plot in Figure 7 with $p_{err}=10^{-6}$, there were 434 hits (or 1.1%) that miss the basket by over $10^{11}$ meters, or roughly the distance between the Earth and Sun! These are not seen in the plot due to the small region shown of only ±10.4 cm. The percentage of misses rises quickly with $p_{err}$, as is shown in Figure 6a. Of course, very large misses stemming from bad computation would not be possible in an actual game due to the limited strength of the robotic arms, but, still, those misses would look spectacular!

This behavior is in stark contrast with that of Man, whose distribution of hits has not a single outlier and is close to the Gaussian (normal) distribution around the center of the hoop. The normal distribution in RPC is not a surprise, because the total deviation from the mean is composed of a large number of small binary errors, which indeed is a feature used in the Hagen’s deduction of the normal distribution [26].

On the other hand, because in the DC binary numbers are represented in a position system where a bit represents a magnitude according to its position in the number, a single bit flip may cause a large error and even a sign flip. In such computation, a single bit-flip error, let alone its further propagation during the computation, is likely to produce a dramatic error in the final result.

## 6. Discussion and Conclusions

The basketball scoring performances of two imaginary mechanical robots were compared as a function of error probability of their circuits: Man which uses an RPC computer and Machine which uses a conventional DC computer. Each robot comprises a hand, which throws the ball, and a black-box system which supplies the momentary distance *D* between the robot and the basketball hoop. The task of the robot is to calculate the force with which it has to push the ball in order to make a score. This implies computing a certain ballistic equation, derived in this study and assumed imprinted (coded) into its computer. With a flawless computer, both robots would score every single time.

However, the main point of this study is the assumption that information-processing circuits in both robots are unreliable and prone to making single-bit-flip errors with a probability $p_{err}$. In practice, such errors can indeed occur due to a number of factors: ionizing radiation, high temperature, electrical noise in the circuits caused by electromagnetic interference (also known as EMI), bad power supply, component aging or for other reasons. Because of that, robots will sometimes miss the goal. The figure of merit for comparing their performances as a function of $p_{err}$ is the scoring probability. The scoring probability was investigated for a set of shooting distances D=1 m, 3 m, 5 m and 7.24 m (the latter being the 3-pointer line). The score is defined as a hit within 10.9 cm from the center of the hoop. The results are as follows.

First, it has been found that Man achieves a significantly higher scoring probability for $p_{err}$ in the range from 0.01% all the way to 10%, while at lower $p_{err}$, both robots converge to the perfect score. While Man scores better, Machine scores more close to the dead center of the hoop for $p_{err}$ up to 0.7%, evaluated at D=3 m distance. Nevertheless, at a higher $p_{err}$, Man is again superior, not only in just making a score, but also in hitting the dead center of the hoop.

Second, it is found that Man’s hits form a Gaussian distribution around the center of the hoop, with a vanishing probability of making large misses even at the highest $p_{err}$ researched (10%). On the other hand, Machine is prone to spectacular misses readily at $p_{err}$ as low as 1 part-per-million. In that case, about 1.1% of all shoots would end up over one hundred million kilometers away from Earth! Of course, because of the limited strength of Machine’s robotic arm, the ball would fall closer, but still it would make a very large miss. This is a strong indication that even a slightly damaged DC-based robot, in a real-life situation, might do crazy things. This could be important for applications where reckless behavior may cause havoc or irreparable damage to the robot or its surroundings and cannot be tolerated; for example, a warehouse picking robot or a surgical robot.

Finally, the complexity of Man’s RPC computer was estimated at about 100 logic gates if it uses a pseudo-random adder with 8-bit LFSR or 65 logic gates plus a quantum entropy source and it uses random adder [20], while it is about 3500 logic gates for the DC computer of Machine.

These findings indicate that the biologically inspired computation requires less hardware for the same task, ensures higher robustness to the hardware malfunction and features a better behaved operation than the digital computation in case of damage. These are all characteristics of high importance for the survivability of living beings and may shed some light on why living beings use pulsed instead of digital computation in their nervous systems.

The results of this study are expected to contribute to the development of biomimetic robots, especially those destined for tasks that could be efficiently performed by the RPC computation, as well as for operation in environments which can impair a robot’s information-processing circuitry. Finally, this study may inspire novel approaches to the operation of prosthetic limbs and their interface with the human nervous system.

## Data Availability

The data that support the findings of this study are available from the author upon reasonable request.
